# Surrounding road density of child care centers in Australia

**DOI:** 10.1038/s41597-022-01172-1

**Published:** 2022-03-31

**Authors:** Chunlei Han, Rongbin Xu, Xiaoyan Wei, Yajuan Zhang, Jiahui Liu, Yuguo Zhang, Tingting Ye, Siwei Wang, Wenhua Yu, Suying Guo, Kun Han, Yimin Ding, Jinfeng Wang, Yuming Guo, Shanshan Li

**Affiliations:** 1 School of Public Health and Management, Binzhou Medical University, Yantai, Shandong 264003 P.R. China; 2 School of Public Health and Preventive Medicine, Monash University, Melbourne, VIC 3004 Australia; 3 Yunnan Provincial Archives of Surveying and Mapping, Kunming, Yunnan 650034 P.R. China; 4 Yunnan Provincial Geomatics Center, Kunming, Yunnan 650034 P.R. China; 5 School of Public Health and Management, Ningxia Medical University, Yinchuan, Ningxia Hui Autonomous Region 750004 P.R. China; 6 School of Geography and Ecotourism, Southwest Forestry University, Kunming, Yunnan 650051 P.R. China; 7 Tangshan Gangxin Technology Development Co., Ltd, Tangshan, Hebei, 063611 P.R. China; 8 Guotai Junan Securities, Shanghai, 200030 P.R. China; 9 School of Economics, Fudan University, Shanghai, 200433 P.R. China; 10 School of software, Tongji University, Shanghai, 200092 P.R. China; 11 State Key Laboratory of Resources and Environmental Information System, Institute of Geographic Sciences and Natural Resources Research, Chinese Academy of Sciences, Beijing, P.R. China; 12 University of Chinese Academy of Sciences, Beijing, P.R. China

**Keywords:** Environmental social sciences, Environmental impact

## Abstract

High surrounding road density could increase traffic-related air pollution, noise and the risk of traffic injuries, which are major public health concerns for children. We collected geographical data for all childcare centers (16,146) in Australia and provided the data on the road density surrounding them. The road density was represented by the child care center’s nearest distance to main road and motorway, and the length of main road/motor way within 100~1000-meter buffer zone surrounding the child care center. We also got the data of PM_2.5_ concentration from 2013 to 2018 and standard Normalized Difference Vegetation Index (NDVI) data from 2013 to 2019 according to the longitude and latitude of the child care centers. This data might help researchers to evaluate the health impacts of road density on child health, and help policy makers to make transportation, educational and environmental planning decisions to protect children from exposure to traffic-related hazards in Australia.

## Abstract (summary)

**Table:** 

Measurement(s)	road density • PM_2.5_ • NDVI
Technology Type(s)	open street map, google map • satellite-, simulation- and monitor-based PM_2.5_ sources • Moderate resolution Imaging Spectroradiometer
Factor Type(s)	ServiceNam • ServiceTyp • ServiceAdd • Suburb • State • Postcode • NumberOfAp • QualityAre • OverallRat • Long_Day_C • Preschool_ • Outside_sc • type • lon_final.x • lat_final.x • PM2.5_2013 • PM2.5_2014 • PM2.5_2015 • PM2.5_2016 • PM2.5_2017 • PM2.5_2018 • NDVIMax_2013 • NDVIMax_2014 • NDVIMax_2015 • NDVIMax_2016 • NDVIMax_2017 • NDVIMax_2018 • NDVIMax_2019 • dismain • dismotorway • main_50 • main_100 • main_300 • main_500 • main_1000 • moto_50 • moto_100 • moto_300 • moto_500 • moto_1000
Sample Characteristic - Organism	child care center
Sample Characteristic - Environment	road density • PM_2.5_ • NDVI
Sample Characteristic - Location	Australia

## Background & Summary

Road transport is one of the main sources of air pollution. Road transport related air pollutants include airborne particulate matter (PM), oxides of sulfur (SO_x_), oxides of nitrogen (NO_x_), carbon monoxide (CO), volatile organic compounds (VOC_s_), polycyclic aromatic hydrocarbons (PAH_s_) and ozone^1,2^, which contributes to 18.4% of total PM emissions worldwide^2^.

Traffic-related air pollution has been associated with adverse health effects, including asthma^3,4^, rhinitis and eczema^4^, cardiovascular disease^5–7^, stroke^8^ and autism^9,10^. Globally, traffic-related PM_2.5_ is responsible for 165,000 deaths per year^11^, 41% of which living within the 0–100 m buffer of the traffic roads^12^. It has been estimated that the premature mortality risk living within 0–100 m buffer of traffic road is 29.5% higher than that of 101–200 m, 179.3% higher than the buffer 201–300 m, and 566% higher than the buffer 301–400 m^12^. It was suggested that people lived within 40 meters of the highways suffered the worst ratings of air quality and a residential separation buffer of 100 meters alongside major highways in the interests of protecting human health^13^. Some other studies recommended that at least 100–150 meters away from the road^14^, 100 and 300 meters perpendicular away from the highway^15,16^. Children are more susceptible to traffic-related air pollution exposure than adults because of their less mature respiratory and immune system, higher breathing rate relative to body size, and more outdoor times^17–19^. Many literatures have reported the harm of traffic-related air pollution to children living or studying in close proximity to major roads^3,20–22^. For example, asthma risk of children attending kindergarten and first grade increased with traffic-related air pollution from roadways by 51% near homes and by 45% near schools^3^. Besides, noise pollution and road injuries are the other two risks of transport beyond air pollution^23–25^. Many studies have documented the adverse health impacts of traffic-related noise among children^26–28^, and road injuries death risk for children^29^. Based on the above reasons, road density is usually used as a proxy for other parameters that are of direct interest, such as actual pollutant concentrations, noise levels and traffic injuries with respect to public health.

As one of the highest rates of motor vehicle owned countries, more than 90% of Australian households have one or more registered motor vehicles^30^. It is of great importance to study the health risk of road density to children in Australia. Although there are some literatures about of road density or distance to road and Children’s health outcomes in some specific areas in Australia^31–33^, studies with national data are scarce. Therefore, a comprehensive national data of child care center is highly needed. Researches based on the presented data may help inform land-use planning agencies to make transport planning decisions, minimize children’s exposure to traffic related hazards. Social policy on the placement of vulnerable populations like children along the main road and motorway will finally improve the environmental health justice.

## Methods

**Fig. 1:**
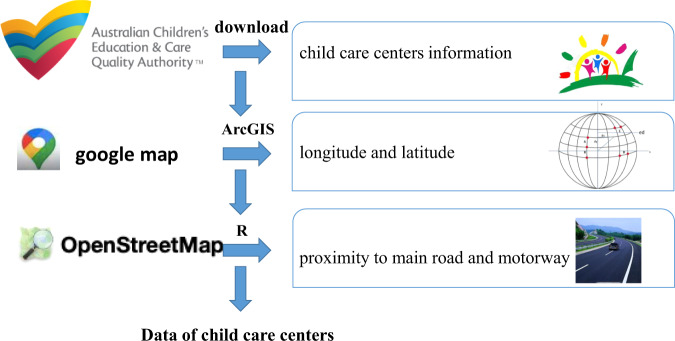
Schematic overview of the process used to get the data.

As described in Fig. 1, the data of registered child care centers were from the website of Australia’s Children’s Education & Care Quality Authority (ACECQA). Data of proximity to main road and motorway of child care centers were from google map using R software (version 3.5.1). Data of vectors of the Australian road network were from Open Street Map (OSM). Briefly, motorways refer to roads with tag “highway = motorway” in OSM, referring to the highest-performance roads within a territory (https://wiki.openstreetmap.org/wiki/Tag:highway%3Dmotorway) In Australia, such kind of roads can be called motorways, freeways, and freeway-like roads, which are divided roads with 2 or 3 lanes in each direction, limited access via interchanges, no traffic lights, and generally 100 or 110 km/h speed limit (https://wiki.openstreetmap.org/wiki/Tag:highway%3Dmotorway). The international equivalents of motorways in other countries have also been described in details elsewhere (e.g., motorways refer to freeway, turnpike, or interstate roads in US) (https://wiki.openstreetmap.org/wiki/Tag:highway%3Dmotorway). Main roads refer to roads with tag “highway = primary” in OSM, normally referring to roads with 2 lanes or more in developed countries, and the traffic for both directions is usually not separated by a central barrier (https://wiki.openstreetmap.org/wiki/Tag:highway%3Dprimary) The equivalents of main roads in Australia have not been defined in OSM, but their equivalents in New Zealand are state highways and strategic local roads, and their equivalents in US are primary highway or arterial road (https://wiki.openstreetmap.org/wiki/Tag:highway%3Dprimary). In the OSM dataset for Australia, motorways and main roads were the two largest types of roads, which can represent exposure to traffic related hazards well.

### How to get the child care center information

We got the child care center information from the website of Australia’s Children’s Education & Care Quality Authority (https://www.acecqa.gov.au/resources/national-registers)on May, 2020. The website provided information of 16,146 approved education and care services and providers, including center based care (long day care, outside school hours care as well as preschool/kindergarten) and family day care. The definitions of long day care is a center-based form of early childhood education and care for children aged 0–6 years. Preschool means childcare centers offering program to prepare children (3–5 years olds) for school. It is called Kindergarten in some states. Outside School Hours Care is for primary school aged children (6–12 years), before and after school (7.30am–9.00am, 3.00pm–6.00 pm), during school holidays and on pupil-free days. Family day care means a flexible form of the early childhood education and care that provided in the private home of carers^34^. The information includes the child care centers name, service type, address, suburb, state, postcode, number of approved children, long-day care or not, preschool or not, outside school hours care or not and overall rating according to the National Quality Framework (NQF). NQF is the national system guided by the ACECQA, introducing a new quality standard in 2012 to improve education and care across long day care, family day care, preschool/kindergarten, and outside school hours care services. Five-point rating scale including significant improvement required, working towards, meeting, exceeding, excellent, and is used to describe the quality of care in individual services across Australia^34^.

### How to get the longitude and latitude

**Fig. 2:**
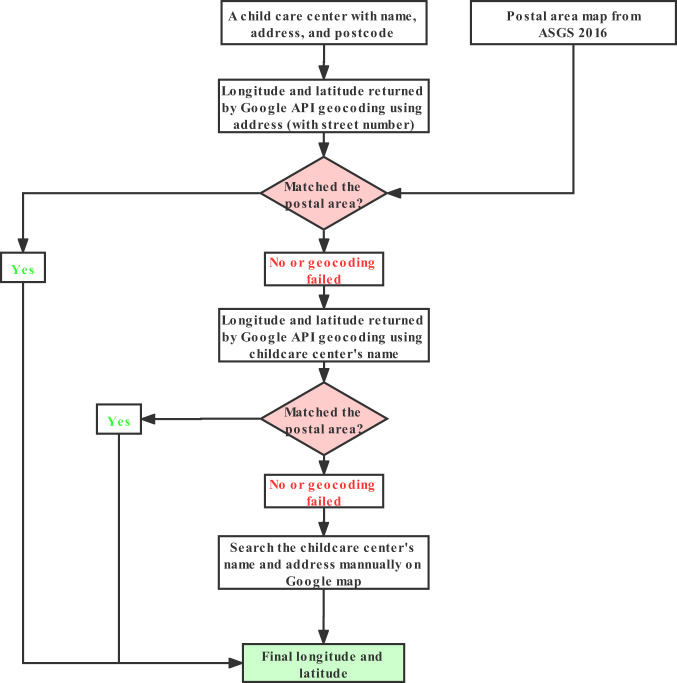
The procedures of determining the longitude and latitude of each childcare center.

We used the Google map API and functions embedded in the “ggmap” R package (version 3.0.0) to transform the address of childcare centers into longitude and latitude. The address and postcode of 6 child care centers were missing, we inputted them manually by searching the name of these child care centers on Google map. Some wrong postcodes (i.e., postcodes do not exist in Australia according to the Australian Statistical Geography Standard 2016 [ASGS 2016]) were corrected manually. Each child care center’s longitude and latitude was determined with the procedures detailed in Fig. 2.

### How to get the proximity to main road and motorway

**Fig. 3:**
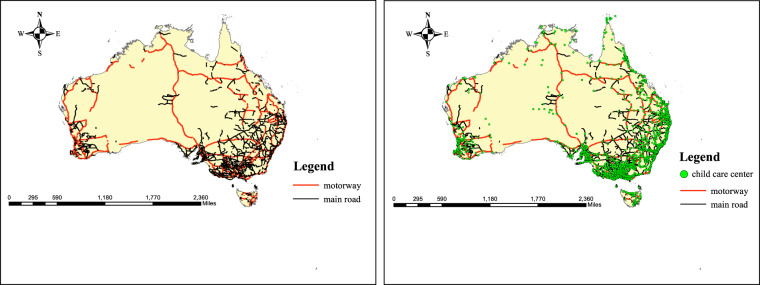
Map of child care centers, main roads and motorways.

1. We downloaded the vectors of the Australian road network from OSM with information of road grade (https://www.openstreetmap.org/#map=4/36.96/104.17).

2. We extracted the road with high-grade and high maximum speed from the dataset.

3. We imported the extracted road and the child care center locations into ArcGIS (version 10.4), and transformed their spherical coordinates into the projected ones by the projection and transformation tool in order to get the distance from every child care center to the nearest road.

4. We calculated the minimum distance between each child care center to the main road by the adjoin analysis tool in the toolbox.

5. We counted respectively the length of road in the buffer zone of 50 meters, 100 meters, 300 meters, 500 meters and 1000 meters of child care centers by the spatial statistical analysis tool (Fig. 3).

### How to get the PM_2.5_ concentration and NDVI data

We derived data of annual average PM_2.5_ concentrations from 2013 to 2018 from a global PM_2.5_ database at 0.01° × 0.01° (approximately 1 km × 1 km) spatial resolution estimated by combining information from satellite-, simulation- and monitor-based sources^35^. Each childcare center was represented by the values of the grid cells where the center located in. We derived data of child cares’ surrounding greenspace represented by Normalized Difference Vegetation Index (NDVI) during 2013–2019 from the Moderate resolution Imaging Spectroradiometer (MODIS) images collected by NASA’s Terra satellite (product number: MOD13A2)^36^. For each child care center in each year, we used the maximum NDVI during summer months [January, February and December (two images per month)] in the grid cell where the center located in to represent the greenspace level.

## Data Records

This section gives details of each data record as listed in Online-only Table 1. Data of Surrounding road density of child care centers, Australia as the Microsoft Excel file can be freely accessed via the Science Data Bank at 10.11922/sciencedb.00728^37^.

## Technical Validation

### Road density of all child care centers

Summary of general characteristics of the final data with 16,146 child care centers and 1,002,600 approved children was listed in Table 1. Median of distance to main road and distance to motorway were 1023.7 (P_25_-P_75_: 361.3–2839) meters and 3997.7 (P_25_-P_75_: 1837.5–10474.8) meters. 4.40% of the child care centers were located in areas with a main road within a distance of 50 meters, and 9.4%, 25.1%, 32.1% and 49.7%within 100, 300, 500 and 1000 meters, respectively. 0.2% of the child care centers were located in areas with a motorway within a distance of 50 meters, and 0.6%, 3.7%, 5.8% and 13.4% within 100, 300, 500 and 1000 meters, respectively. 4.8% of the number of approved children in child care center located within 50 meters surrounding the main road, 10.1%, 25.7%, 32.8% and 50.6% within 100, 300, 500 and 1000 meters. Correspondingly, 0.2% of the number of approved children in child care center located within 50 meters surrounding the motorway, 0.7%, 4.0%, 6.4% and 14.7% within 100, 300, 500 and 1000 meters. From 2013 to 2018, mean and standard deviation of annual average PM_2.5_ concentration were 5.01ug/m^3^ and 1.66 ug/m^3^ (with the minimum of 1.96 ug/m^3^ and maximum of 9.69 ug/m^3^). NDVI (standard deviation) were 0.47 and 0.14 averagely from 2013 to 2019.

**Table 1:** Summary of data of child care centers.

Variable	Number of child care centers (%)	Number of approved children (%)	Mean	SD	Min	P_25_	Median	P_75_	Max	PM_2.5_ (SD)	NDVI(SD)
Dismain	—	—	5738.92	23676.6	0.03	361.3	1023.7	2839	1700709	—	—
Dismotorway	—	—	59329.62	224788.6	0.29	1837.5	3997.7	10474.8	2771484	—	—
Main50	708 (4.4)	47894 (4.8)	3.51	19.02	0	0	0	0	363.7	5.51 (1.66)	0.44 (0.14)
Main100	1514 (9.4)	100809 (10.1)	21.96	76.86	0	0	0	0	793.4	5.43 (1.63)	0.45 (0.14)
Main300	4048 (25.1)	257879 (25.7)	251.10	513.78	0	0	0	19.8	5199.2	5.35 (1.64)	0.45 (0.14)
Main500	5179 (32.1)	329041 (32.8)	458.2	814.78	0	0	0	837.6	8009.4	5.35 (1.65)	0.45 (0.14)
Main1000	8018 (49.7)	506826 (50.6)	1626	2216.75	0	0	0	2934	17321	5.37 (1.65)	0.45 (0.13)
Motor50	33 (0.2)	2268 (0.2)	0.22	5.37	0	0	0	0	198.6	6.48 (0.96)	0.43 (0.15)
Motor100	102 (0.6)	7381 (0.7)	1.44	20.91	0	0	0	0	691.1	6.04 (1.16)	0.49 (0.13)
Motor300	590 (3.7)	40137 (4.0)	39	226.85	0	0	0	0	3935	5.73 (1.41)	0.49 (0.15)
Motor500	932 (5.8)	64468 (6.4)	88.23	406.86	0	0	0	0	6081	5.74 (1.40)	0.49 (0.14)
Motor1000	2168 (13.4)	147100 (14.7)	432.4	1289.26	0	0	0	0	18478.7	5.61(1.52)	0.47 (0.14)

**Notes:** Dismain: distance to main road. Dismotorway: distance to motorway. Main50~Main1000: length of main road in 50-meter (~1000-meter) buffer zone, or within 50 meters (~1000 meters) surrounding the child care center. Motor50~Motor1000: length of motorway in 50-meter (~1000-meter) buffer zone, or within 50 meters (~1000 meters) surrounding the child care center. SD: standard deviation. Min: minimum. P_25_: the 25^th^ percentile. P_75_: the 75^th^ percentile. Max: maximum. PM_2.5_: annual average PM_2.5_ concentration from 2013 to 2018, ug/m^3^. NDVI: maximum Normalized Difference Vegetation Index in summer months from 2013 to 2019. Units of distance and length are meters.

### Road density by state, NQF and school type

**Fig. 4:**
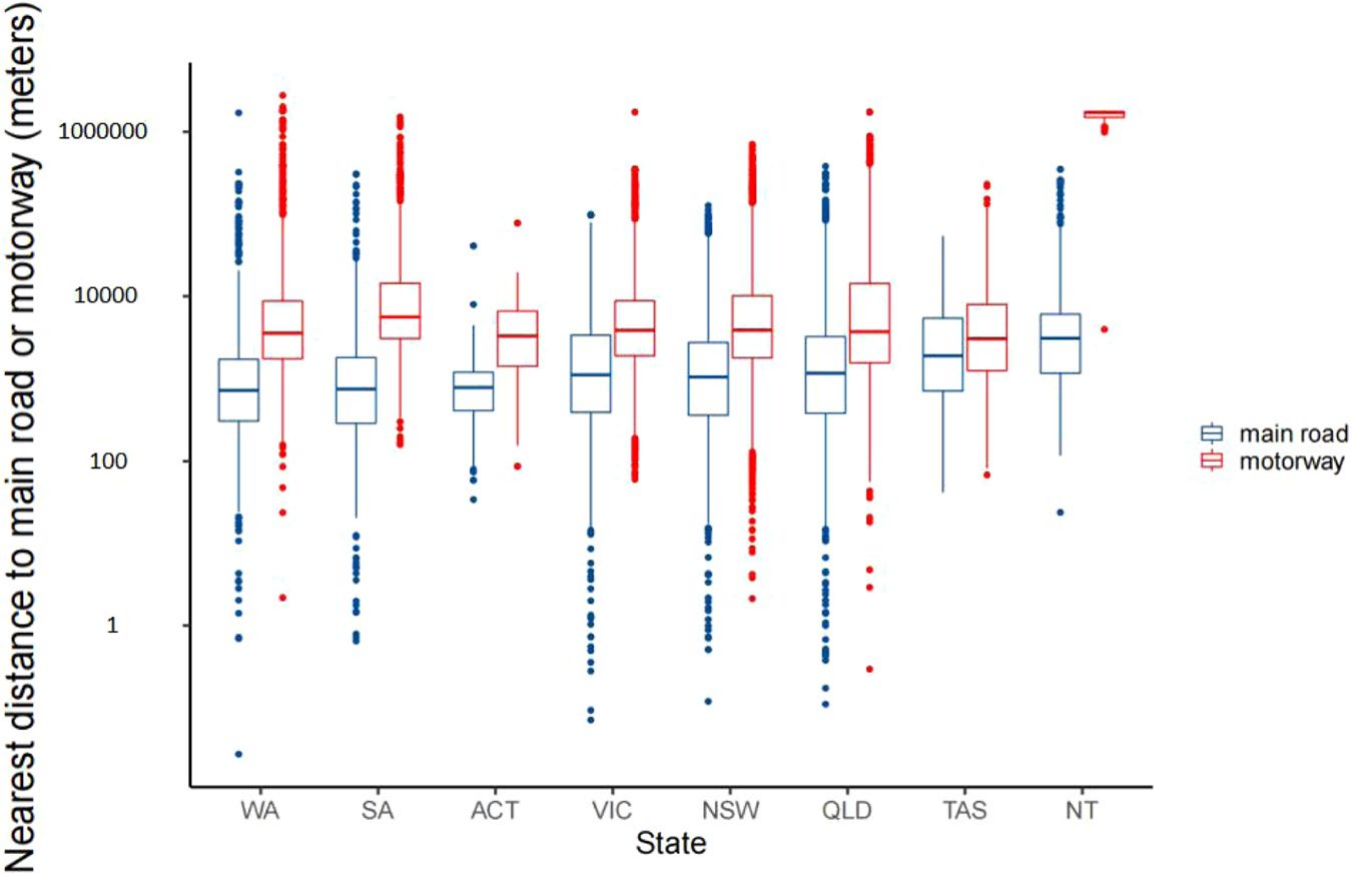
Nearest distance to main road and motorway of the child care center by state. Note: Western Australia (WA), South Australia (SA), Australian Capital Territory (ACT), Victoria (VIC), New SouthWales (NSW), Queensland (QLD), Tasmania (TAS), Northern Territory (NT).

**Fig. 5:**
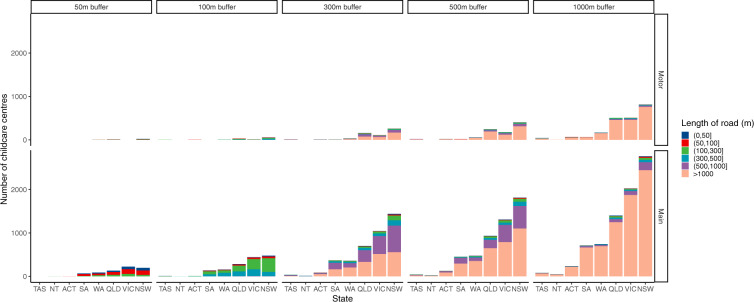
Length of main road and motorway within 100 to 1000 meters buffers around the child care center by state. Note: Tasmania (TAS), Northern Territory (NT), Australian Capital Territory (ACT), South Australia (SA), Western Australia (WA), Queensland (QLD), Victoria (VIC), New SouthWales (NSW).

Figure 4 showed nearest distance to main road and motorway of the child care center by state. Nearest median distances to main road and motorway are 715.66 meters (P_25_~P_75_: 303.20~1707.30) in Western Australia and 3040.18 meters (P_25_~P_75_: 1249.23~7954.96) in Tasmania. Figure 5 showed lengths of main road and motorway within 50 to 1000 meters buffers around the child kid center by state, among which the longest main road and motorway were New South Wales, Victoria, and Queensland.

**Fig. 6:**
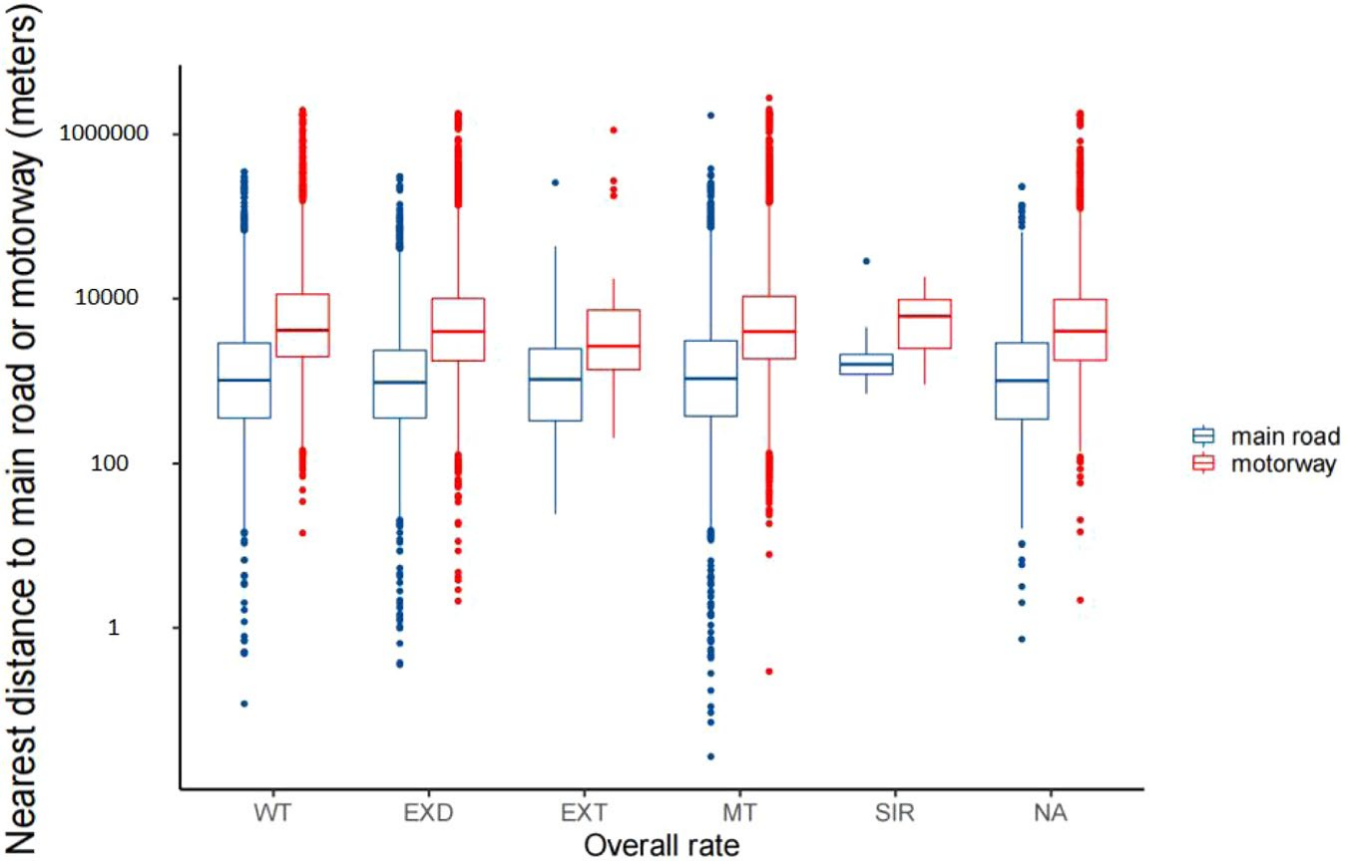
Nearest distance to main road and motorway of the child care center by overall rating. **Note:** SIR: Significant Improvement Required, WT: Working Towards, MT: Meeting, EXD: Exceeding, EXT: Excellent,. NA: missing data.

**Fig. 7:**
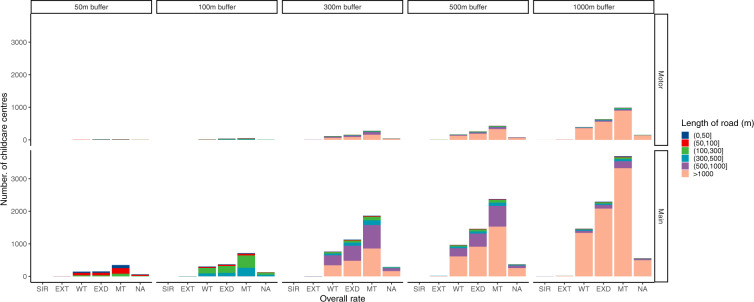
Length of main road and motorway within 100 to 1000 meters buffers around the child care center by overall rating. **Note:** SIR: Significant Improvement Required, WT: Working Towards, MT: Meeting, EXD: Exceeding, EXT: Excellent,. NA: missing data.

Nearest distance to main road and motorway and lengths of main road and motorway within 50 to 1000 meters by overall rating of the child care according to NQF were shown in Figs. 6 and 7. There was no clear trend that the nearest distance to main road or motorway vary by overall rating level of the child care. Childcare centers with the longest main road and motorway were those of Meeting and Exceeding National Quality Standard.

**Fig. 8:**
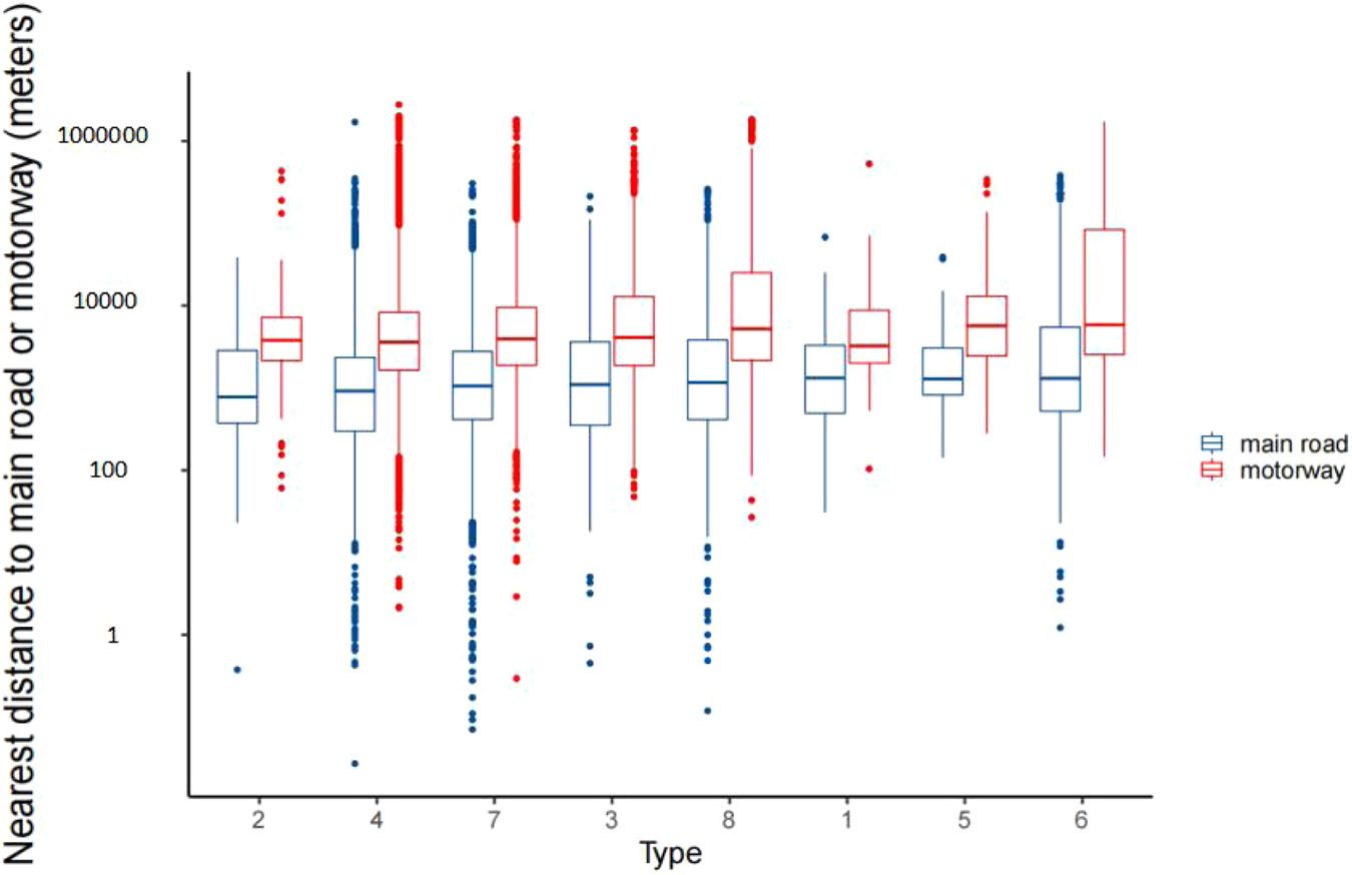
Nearest distance to main road and motorway of the child care center by school type.

**Fig. 9:**
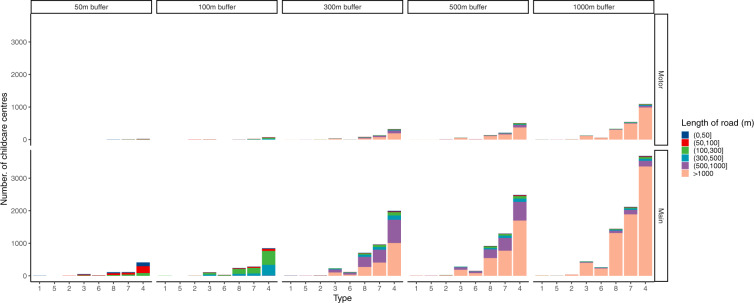
Length of main road and motorway within 100 to 1000 meters around the child care center by school type.

Figures 8 and 9 showed the nearest distance to main road and motorway, and the length of main road and motorway within 50 to 1000 meters by school type (See Table 2 of child care center types symbol). Median nearest distances to main road and motorway are 776.24 meters (P_25_~P_75_: 705.58~5409.84) of child care centers of long day care and preschool (type 2) and 3237.95 meters (P_25_~P_75_: 1249.23~7954.96) of child care centers of long day care, preschool and outside school hours care (type1). The longest main road and motorway located within 50 to 1000 meters near child care centers were those of only long day care (type 4).

**Table 2:** Types of child care centers symbol.

Type	Long day care	Preschool	Outside school hours care	Number	Proportion (%)
1	yes	yes	yes	36	0.22
2	yes	yes	no	69	0.43
3	yes	no	yes	905	5.61
4	yes	no	no	7004	43.38
5	no	yes	yes	37	0.23
6	no	yes	no	611	3.78
7	no	no	yes	4350	26.94
8	no	no	no	3134	19.41

Types of child care centers are symbolled 1–8 based on whether the center is long day care, preschool or outside school hours care. Among all types of child care centers, type of only long day care is the largest group (7004), representing about 43.28% of all child care centers. The details are listed in Table 2.

## Usage Notes

The presented data allow for spatial aggregations of the child care centers, the proximity to the main road and motorway. We have downloaded the registered child care centers and Australian road network till May, 2020. However, the data will update with time. We comprehensively consider the impacts of environmental variables in our data, such as PM_2.5_ and NDVI, other air pollutants and noise pollution were not available because of the data missing.

By linking the longitude and latitude of child care centers, this data could be used to analyze the health risk and disease burden for children from exposure to traffic or health benefit from NDVI surrounding the main road or motorway by combining the children’s mortality or morbidity data in Australia. It could be used to describe the overall situation and compare the traffic related air or noise pollution in different study areas by linking other air pollutants or traffic noise data. Data of traffic density could be added to our data, such as the numbers and types of fleet or main road or motorway, in order to compare rural areas with urban areas traffic or traffic-related health risks. This dataset’s utility can be further enriched if other relevant pieces of information could be added, such as census data (e.g. population density), land use, and socioeconomic and demographic information for areas within specified buffers around the childcare centers. We provided the data in the format of excel, making it easy to be further analyzed in statistical software like R, Stata, SAS and SPSS.

The implementation of the data may help better design and redistribution of child care centers, and assist transportation, infrastructure, environmental planning and socioeconomic and demographic health equity for the governments. For parents of children under 12 to avoid studying in areas with the traffic related air pollution in study areas. The results may provide assistance to improve vehicle technology and to change travelling behaviors, including the increased use of public transport and active travel to reduce traffic emissions.

## Supplementary information

dataset 1

## Data Availability

All of the custom code used for the generation and analysis of this dataset is publicly available at the diyiyonghu GitHub repository at https://github.com/diyiyonghu/analysis-code.git.

## Online-only Table

**Online-only Table 1:** Records of child care center data.

Variable	Description	Type	Range or levels	How measured	Units/formats/level coding	The number and proportion of missing values n(%)
V1	Serial number	factor	1~16,146	identified	—	0 (0)
ServiceNam	Name	factor	—	identified	—	0 (0)
ServiceTyp	Type	factor	16,146 individuals	identified	Center-based-care, family day care	0 (0)
ServiceAdd	Address	factor	16,146 individuals	identified	—	6 (0.04)
Suburb	Suburb belonged to	factor	3,869 suburbs	identified	—	6 (0.04)
State	State located in	factor	8 states	identified	New SouthWales (NSW), Victoria (VIC),	0 (0)
					Queensland (QLD), Western Australia (WA), South Australia (SA),	
					Tasmania (TAS),	
					Australian Capital Territory (ACT),	
					Northern Territory (NT)	
Postcode	Postcode	integer	—	identified	—	6 (0.04)
NumberOfAp	Number of approved children	numeric	0~414	identified	person	526 (3.26)
QualityAre	Quality level according to the National Quality Standard	factor	three levels	identified	Exceeding NQS, Meeting NQS, Working Towards NQS	1096 (6.79)
OverallRat	Overall rating according to the National Quality Standard	factor	five levels	identified	SIR: significant improvement required, WT: working towards, MT: meeting, EXD: exceeding, EXT: excellent	1096 (6.79)
Long_Day_C	Long day care	factor	yes, no	identified	yes, no	0 (0)
Preschool_	Preschool	factor	yes, no	identified	yes, no	0 (0)
Outside_sc	Outside school hours care	factor	yes, no	identified	yes, no	0 (0)
type	Type of care	integer	1~8	defined	We devide all the child care centers into 8 groups according to whether it offers long day care, preschool or outside school hours care. Details are in Table 3.	0 (0)
lon_final.x	longitude	factor	105.7~153.6	identified	—	0 (0)
lat_final.x	latitude	factor	−43.315~−9.598	identified	—	0 (0)
PM2.5_2013~PM2.5_2018	annual average PM_2.5_ concentration from 2013 to 2018	numeric	1.76~11.39	measured	ug/m^3^	PM2.5_2013~ PM2.5_2016: 0 (0)
						PM2.5_2017~ PM2.5_2018:
						80 (0.50)
NDVIMax_2013~NDVIMax_2019	the annual average NDVI of January, February and December from 2013 to 2019	numeric	−0.29~0.99	measured	—	2034 (12.60)
PM2.5	the mean of PM2.5_2013~ PM2.5_2018	numeric	1.96~9.69	measured	ug/m^3^	80 (0.50)
NDVI	the mean of ndviMax_2013~ndviMax_2019	numeric	−0.19~0.89	measured	—	2034 (12.60)
dismain	Distance to main road	numeric	0~1700709	measured	meter	0 (0)
dismotorway	Distance to motorway	numeric	0~2771484	measured	meter	0 (0)
main_50	length of main road in 50-meter buffer zone	numeric	0~363.7	measured	meter	0 (0)
main_100	length of main road in 100-meter buffer zone	numeric	0~793.4	measured	meter	0 (0)
main_300	length of main road in 300-meter buffer zone	numeric	0~5199.2	measured	meter	0 (0)
main_500	length of main road in 500-meter buffer zone	numeric	0~8009.4	measured	meter	0 (0)
main_1000	length of main road in1000-meter buffer zone	numeric	0~17321	measured	meter	0 (0)
moto_50	length of motorway in 50-meter buffer zone	numeric	0~198.6	measured	meter	0 (0)
moto_100	length of motorway in 100-meter buffer zone	numeric	0~691.1	measured	meter	0 (0)
moto_300	length of motorway in 300-meter buffer zone	numeric	0~3935	measured	meter	0 (0)
moto_500	length of motorway in 500-meter buffer zone	numeric	0~6081	measured	meter	0 (0)
moto_1000	length of motorway in 1000-meterbuffer zone	numeric	0~18478.7	measured	meter	0 (0)

**Notes:** PM_2.5_: annual averagePM_2.5_ concentration from 2013 to 2018, ug/m^3^. NDVI: maximum Normalized Difference Vegetation Index of summer months from 2013 to 2019.
